# Association Between Alpha-1-Acid Glycoprotein and Non-Alcoholic Fatty Liver Disease and Liver Fibrosis in Adult Women

**DOI:** 10.3390/metabo15040280

**Published:** 2025-04-17

**Authors:** Yansong Fu, Siyi Zhang, Xin Zeng, Hong Qin

**Affiliations:** 1 Department of Nutrition and Food Hygiene, Xiangya School of Public Health, Central South University, Changsha 410031, China; 226901016@csu.edu.cn (Y.F.); 246901011@csu.edu.cn (X.Z.); 2 Xiangya School of Medicine, Central South University, Changsha 410031, China; 8303211327@csu.edu.cn

**Keywords:** alpha-1-acid glycoprotein, nonalcoholic fatty liver disease, liver fibrosis, cross-sectional study, controlled attenuation parameter

## Abstract

**Background:** Alpha-1-acid glycoprotein (AGP) is a glycoprotein synthesized mainly by the liver. Nonalcoholic fatty liver disease (NAFLD) and liver fibrosis (LF) are associated with metabolic disorders. The aim of this study was to examine the potential correlation between AGP and both NAFLD and LF. **Methods:** The data were derived from the 2017–2023 National Health and Nutrition Examination Survey (NHANES). The linear association between AGP and NAFLD and LF was examined by multivariate logistic regression models. Non-linear relationships were described by fitting smoothed curves and threshold effect analysis. Subgroup analysis was also performed to assess potential regulatory factors. **Results:** The study included 2270 females. AGP was found to be significantly and positively associated with NAFLD [OR = 12.00, 95% CI (6.73, 21.39), *p* < 0.001] and LF [OR = 2.20, 95% CI (1.07, 4.50), *p* = 0.042]. Furthermore, the association between AGP and NAFLD was significantly different in the diabetic subgroup (*p* < 0.05 for interaction). Additionally, we found an inverted U-shaped relationship between AGP and controlled attenuation parameter (CAP), with an inflection point at 1.20 g/L. **Conclusions:** We found a significant positive correlation between AGP and both NAFLD and LF, and there was an inverted U-shaped relationship between AGP and CAP.

## 1. Introduction

Non-alcoholic fatty liver disease (NAFLD) has become one of the most prevalent chronic liver diseases worldwide, and it is considered the hepatic expression of metabolic syndrome [1]. Epidemiological data indicate that the global prevalence of NAFLD ranges from 25 to 30%, and up to 40% in certain developed countries [2]. With the continuous increase in global obesity and metabolic syndrome, NAFLD has become a major public health problem.

NAFLD is a complex disease, the metabolism of which involves multiple substances and pathophysiological processes [3], such as insulin resistance (IR), oxidative stress, abnormalities in lipid metabolism, inflammatory responses, and hepatocellular steatosis [4,5]. In addition, genetic and environmental effects on gene expression are also reflected in NAFLD. It is characterized by an excessive accumulation of toxic lipids in the liver [1]. From NAFLD to non-alcoholic steatohepatitis (NASH) to liver fibrosis (LF), cirrhosis, and even hepatocellular carcinoma (HCC), NAFLD can present different stages and severity. Current studies demonstrate that approximately 15–20% of NAFLD patients progress to NASH [6]. The metabolic syndrome may cause severe fibrosis in patients with NASH. Approximately 20% of NASH patients will eventually develop LF [7].

Alpha-1-acid glycoprotein (AGP) is an acute-phase glycoprotein synthesized mainly by the liver, which plays an important regulatory role in inflammation and metabolic disorders [8,9]. Serum concentrations of AGP rapidly respond to inflammatory states, and cytokines such as IL-1, IL-6, IL-8, and TNF-α can regulate the AGP gene expression [10,11]. Existing studies have shown that AGP can influence the progression of hepatic inflammatory responses by regulating the expression and activity of inflammatory factors [12,13]. In addition, studies have found that AGP can inhibit neutrophil adhesion and migration, which may reduce inflammatory responses [14], regulate liver lipid metabolism by affecting lipid transport and fatty acid oxidation processes [12,15], and potentially help reduce liver damage by regulating oxidative stress [16].

Furthermore, AGP contains multiple N-glycosylation sites. In liver cirrhosis, AGP levels typically increase, and its glycosyl structure may change [17,18], possibly linked to impaired hepatocyte surface glycoprotein receptors and incomplete glycoprotein production [19]. Mooney et al. proposed that AGP glycosylation is influenced by the degree of fibrosis and that its fucose level may be a predictor of the level of fibrosis within the liver [20]. Experimental studies by Ozeki T et al. demonstrated that AGP injection in rats with chronic liver injury induced by carbon tetrachloride led to increased hepatic fibers and hydroxyproline content in liver collagen [21], suggesting AGP may act as an accelerator of liver fibrosis in chronic hepatitis [22].

Previous studies have found that the detection of AGP levels can be used as a diagnostic tool to determine inflammation [23]. AGP has diagnostic significance for HCC and cirrhosis [24,25,26,27], in which the combined use of alpha-fetoprotein (AFP) and AGP assays may be useful for early diagnosis of HCC patients with cirrhosis presenting [28,29].

However, despite these findings, the specific relationship between AGP and earlier stages of liver disease, particularly NAFLD and liver fibrosis in the absence of cirrhosis, remains poorly understood. Furthermore, the possible non-linear relationships and effect modifiers in this association have not been explored. Our study aims to address these gaps by examining the relationship between AGP and both NAFLD and LF in a nationally representative sample of adult women, thereby providing insights into the mechanisms of liver disease progression. At the same time, we attempted to identify novel disease markers.

## 2. Materials and Methods

### 2.1. Study Population

The National Health and Nutrition Examination Survey (NHANES) represents a comprehensive nationwide health assessment program in the United States. This ongoing surveillance initiative incorporates extensive questionnaires, clinical assessments, and biochemical measurements to evaluate the well-being of community-dwelling Americans. The program is administered through collaborative efforts between the National Center for Health Statistics and the Centers for Disease Control and Prevention.

**Figure 1:**
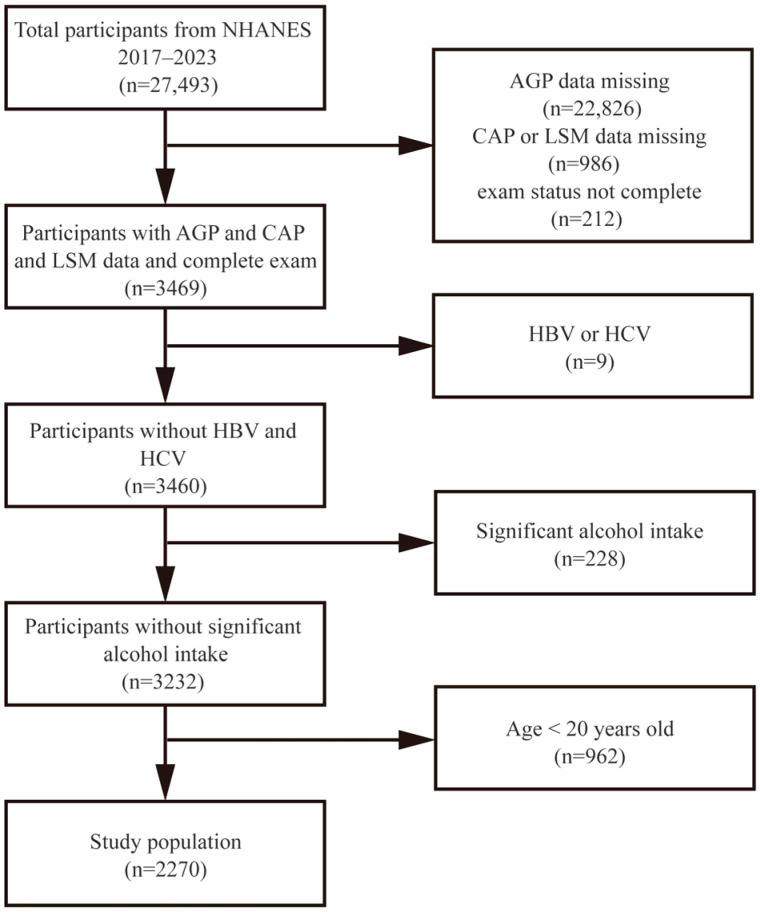
Flowchart of participant selection. NHANES, National Health and Nutrition Examination Survey; AGP, alpha-1-acid glycoprotein; CAP, controlled attenuation parameter; LSM, liver stiffness measurement.

The current analysis drew from a pool of 27,493 survey respondents. Subsequently, 22,826 cases lacking AGP data, 986 cases with missing controlled attenuation parameter (CAP) or liver stiffness measurement (LSM) data, and 212 cases with incomplete examination status were removed from consideration, leaving 3469 participants with complete measurements. An additional 9 participants were excluded due to viral hepatitis infection, as indicated by positive serological markers for hepatitis B surface antigen or hepatitis C antibody. In the context of NAFLD assessment, we further eliminated 228 subjects reporting regular heavy alcohol consumption (defined as daily intake of 4–5 or more alcoholic beverages). After excluding 962 individuals under 20 years of age, the final analytical cohort comprised 2270 participants. A detailed participant selection diagram is presented in Figure 1.

### 2.2. Laboratory Analysis

Data for AGP were only available from female participants aged 1–5 years and 12–49 years, reflecting the specific biomarker collection protocol established by the National Center for Health Statistics. AGP quantification was performed using a Tina-quant Gen.2 immunoturbidimetric procedure (Roche Diagnostics, Indianapolis, IN, USA) on a Roche Cobas platform (Roche Diagnostics, Indianapolis, IN, USA). This method measures immune complex formation between AGP and specific antibodies through turbidity assessment at 340 nm wavelength. This dataset-specific limitation provided us with a valuable opportunity to conduct a focused analysis on adult women and NAFLD.

### 2.3. Hepatic Assessment

Liver evaluation was conducted using vibration-controlled transient elastography (VCTE) (FibroScan^®^, Echosens, Paris, France) with either M or XL probes (Echosens, Paris, France) based on body habitus. As such, TE has become the non-invasive test of choice in most liver clinics around the world [30]. VCTE simultaneously measures CAP for hepatic steatosis quantification and LSM for fibrosis assessment. To ensure the highest data quality and reliability, we implemented strict validation protocols for hepatic measurements. Valid examinations required three critical criteria: (1) a minimum 3 h fasting period, (2) at least ten successful measurements, and (3) an interquartile range/median ratio below 30%. Measurements that did not satisfy these rigorous standards were excluded to minimize potential measurement errors and maintain the precision of our analysis, in accordance with best practices in transient elastography. We defined NAFLD as CAP ≥ 274 dB/m and LF as LSM ≥ 8.0 kPa [31,32].

### 2.4. Covariates

Based on the previous reports, the NAFLD-associated covariates were included, such as age, race, education level, marital status, ratio of family income to poverty (PIR), body mass index (BMI), smoking status, diabetes, hypertension, cardiovascular disease (CVD), and total cholesterol. Hypertension was defined as systolic blood pressure ≥130 mmHg and/or diastolic blood pressure ≥80 mmHg on ≥3 occasions. Moreover, participants who answered “yes” to the questions: “Are you now taking prescribed medicine for high blood pressure?” or “Ever told you had high blood pressure?” were also defined as having hypertension. Diabetes was defined as a positive response to the question: “Doctor told you have diabetes?” and/or “Are you now taking insulin?” and/or “Are you now taking diabetic pills to lower blood sugar?” Additionally, participants who achieved one or more of the following conditions were diagnosed with diabetes: glycohemoglobin ≥ 6.5%, fasting glucose ≥ 7 mmol/L. A history of CVD was defined as a positive response to the question: “Has a doctor or other health professional ever told you that you had congestive heart failure?” or “Has a doctor or other health professional ever told you that you had coronary heart disease?” or “Has a doctor or other health professional ever told you that you had angina?” or “Has a doctor or other health professional ever told you that you had heart attack?” or “Has a doctor or other health professional ever told you that you had stroke?” PIR can be divided into three groups: PIR ≤ 1.3 representing low income, 1.3 < PIR ≤ 3.5 representing middle income, and PIR > 3.5 representing high income [33].

### 2.5. Statistical Methodology

We used weighted data analysis throughout this study. Specifically, we used the svydesign function to create a survey design object incorporating the appropriate sampling weights (WTMEC2YR), primary sampling units (SDMVPSU), and strata variables (SDMVSTRA) following NHANES analytical guidelines. This approach addresses the potential for variance underestimation that can occur when applying standard statistical methods to complex survey data, thereby providing more accurate effect estimates and confidence intervals.

In the descriptive analysis, we presented continuous variables as arithmetic means with standard error means (95% CI), while categorical variables were summarized as frequencies and proportions. To evaluate the associations between AGP and hepatic outcomes (NAFLD and LF), we constructed sequential weighted logistic regression models with increasing levels of adjustment: an unadjusted base model (Model 1); a demographic-adjusted model incorporating age and race (Model 2); and a comprehensive model further controlling for educational level, marital status, PIR, BMI, smoking, diabetes, hypertension, history of CVD, total cholesterol (Model 3). Non-linear relationships were explored using smoothed curve fitting and threshold effects analysis. The investigation utilized two-piecewise linear regression to evaluate potential inflection points in AGP-CAP/LSM relationships. Subgroup analysis was also performed to assess potential regulatory factors. Analyses were performed using R (version 4.2.0, R Foundation for Statistical Computing, Vienna, Austria) and EmpowerRCH platforms (version 4.2, X&Y Solutions, Boston, MA, USA), with statistical significance set at *p* < 0.05.

## 3. Results

### 3.1. Baseline Characteristics of Participants

A total of 2270 adult female participants were selected for the study, with a mean age of 35.01 ± 8.43 years. Among all participants, 38.59% were non-Hispanic white, 92.56% were diabetes-free, and 98.02% had no history of CVD. The mean BMI was found to be 29.76 ± 8.14 kg/m^2^. The average levels of AGP, CAP, and LSM were 0.79 ± 0.24 g/L, 246.42 ± 61.47 dB/m, and 5.02 ± 2.92 kPa, respectively. The prevalence of NAFLD was 31.15%, and the prevalence of LF was 6.56%. The participants were categorized into three groups based on tertiles of AGP levels, namely Tertile 1 (AGP < 0.673 g/L, n = 757), Tertile 2 (0.673 g/L ≤ AGP ≤ 0.879 g/L, n = 755), and Tertile 3 (AGP > 0.879 g/L, n = 758).

Table 1 shows the basic information of the participants with CAP or LSM as a column-stratified variable. When CAP is a column stratification variable, differences in BMI, smoking status, diabetes, hypertension, and CVD among participants were statistically significant (*p* < 0.05). In comparison with the non-NAFLD group, individuals in the NAFLD group demonstrated a higher propensity for diabetes, hypertension, and CVD. Participants with NAFLD exhibited significantly higher BMI and AGP levels.

When LSM is a column stratification variable, significant differences were observed in BMI, diabetes, hypertension, CVD, and total cholesterol (*p* < 0.05). Those with LF were more likely to have diabetes, hypertension, and CVD compared to the normal population. Participants in the LF group exhibited higher BMI, PIR, and AGP levels.

### 3.2. Association Between AGP and NAFLD

By adjusting for different covariates, three models were used to assess the effect of AGP on NAFLD (Table 2). We found a significant difference in AGP in participants with NAFLD compared to those without NAFLD [OR = 14.95, 95% CI (8.42, 26.53), *p* < 0.001]. After adjusting for all covariates, the difference remained significant [OR = 12.00, 95% CI (6.73, 21.39), *p* < 0.001], with a positive correlation between AGP and the degree of hepatic steatosis. When AGP was considered in tertiles, participants in Tertile 3 were significantly more likely to have NAFLD compared to Tertile 1 [OR = 4.87, 95% CI (3.67, 6.45), *p* < 0.001].

### 3.3. Association Between AGP and LF

Table 3 shows the results of the multivariate logistic regression model between AGP and LF. We found a significant difference in AGP in participants with LF compared to normal subjects [OR = 3.62, 95% CI (1.67, 7.85), *p* = 0.002]. After adjusting for all covariates, the difference remained significant [OR = 2.20, 95% CI (1.07, 4.50), *p* = 0.042], with a positive correlation between AGP and the degree of LF.

### 3.4. Non-Linear Relationship Between AGP and CAP

**Figure 2:**
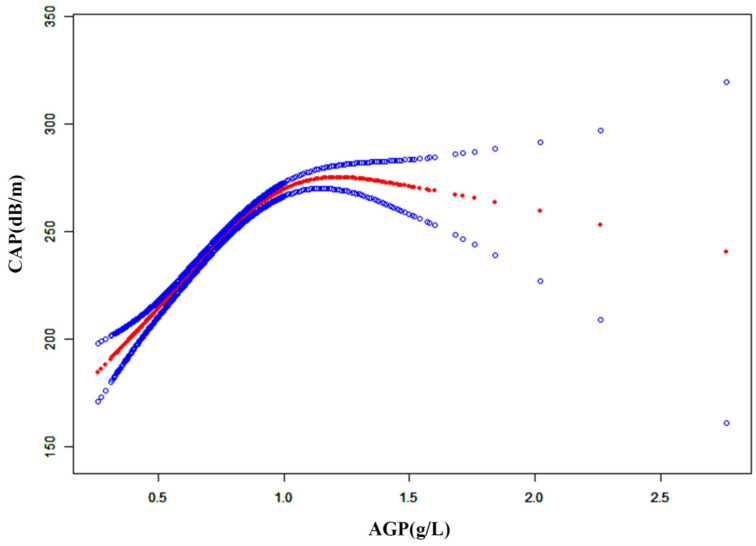
The association between AGP and CAP. The solid red line represents the smooth curve fit between variables. Blue bands represent the 95% confidence interval from the fit.

An inverted U-shaped relationship was found between AGP and CAP using smooth curve fitting and generalized additive modeling (Inflection point: 1.20) (Table 4 and Figure 2). When AGP ≤ 1.20 g/L, the relationship was positive [β = 102.26, 95% CI (91.46, 113.06), *p* < 0.001]. Conversely, when AGP > 1.20 g/L, a negative correlation was observed [β = −60.07, 95% CI (−99.09, −21.05), *p* = 0.003].

### 3.5. Subgroup Analysis

In order to investigate the association of AGP with NAFLD and LF in various population situations, subgroup analysis was performed (Table 5). The association between AGP and NAFLD was significantly different in the diabetic subgroup (*p* < 0.05 for interaction). The association between AGP and LF was significantly different in the subgroups of education level and marital status (*p* < 0.05 for interaction). In addition, a significant positive correlation was identified between AGP levels and both NAFLD and LF in all subgroups.

## 4. Discussion

In this study of adult women, AGP levels were positively correlated with NAFLD and LF. There was an inverted U-shaped relationship between AGP and CAP, with an inflection point of 1.20 g/L. Subgroup analysis suggested that the association between AGP and NAFLD was significantly different in the diabetic subgroup. To our knowledge, this is the first cross-sectional study investigating the association of AGP levels with NAFLD and LF.

Our study population’s relatively young mean age (35.01 years) with a high NAFLD prevalence (31.15%) warrants explanation. Recent studies have shown NAFLD prevalence reaching up to 57.4% in morbidly obese young adults aged 18–35 years, with overall prevalence rising from 9.6% in 1988–1994 to 24% in 2005–2010 [34]. Several factors specific to our study population may contribute to this observation: (1) the exclusion of women over 49 years due to NHANES AGP data availability constraints; (2) the high prevalence of obesity in our cohort (mean BMI 29.76 kg/m^2^); and (3) the use of CAP ≥ 274 dB/m as the NAFLD diagnostic threshold, which has shown high sensitivity.

The focus of our study on adult women provides an opportunity to consider sex-specific aspects of the AGP-liver disease relationship. Epidemiological studies have consistently reported sex-based differences in NAFLD prevalence and progression, with premenopausal women showing lower rates compared to men, but this advantage diminishes after menopause [35,36]. This suggests potential protective effects of estrogens against NAFLD development and progression. Previous research has shown that estrogens can influence both inflammatory responses and glycoprotein expression patterns [37,38,39]. However, the deeper interactions between estrogen, AGP, and NAFLD still need to be further investigated.

Our findings contribute to the growing evidence regarding AGP’s diagnostic potential. In a Korean case study, researchers found that serum asialo-alpha-1-acid glycoprotein was an independent risk factor for the prediction of cirrhosis, and its sensitivity and specificity for the detection of cirrhosis were 79.2% and 64.6%, respectively [24]. Our results extend these findings to earlier stages of liver disease, suggesting that AGP has the potential to be a novel non-invasive diagnostic marker for NAFLD and LF. This is particularly important as the biopsy rate in NAFLD patients is currently low [40], and there is no unique biomarker that is acknowledged to meet diagnostic requirements sufficiently [41]. Furthermore, compared to other possible markers such as serum endotrophin, our results indicate that AGP measurements may also be indicative of the degree of LF [42].

The association between AGP and LF (OR = 2.20, 95% CI [1.07, 4.50]) was indeed lower than that between AGP and NAFLD (OR = 12.00, 95% CI [6.73, 21.39]). This difference is likely attributable to several factors: (1) the substantially smaller sample size of the LF group (6.56% prevalence) compared to the NAFLD group (31.15% prevalence), resulting in wider confidence intervals and potentially reduced statistical power; (2) the multifactorial etiology of liver fibrosis, which involves complex interactions between various inflammatory mediators, fibrogenic factors, and genetic determinants beyond the influence of AGP alone; and (3) the possibility that AGP may play a more direct role in hepatic steatosis development compared to fibrogenesis.

Our findings demonstrated an inverted U-shaped relationship between AGP and CAP. When AGP ≤ 1.20 g/L, AGP was a risk factor for hepatic steatosis, and AGP and CAP were positively correlated. The pathogenesis of NAFLD is a metabolic abnormality involving excessive accumulation of triglycerides, a chronic low-grade inflammatory response [43,44]. First, body fat accumulation in women is associated with elevated AGP [45]. In addition, AGP levels and the development of inflammation in NAFLD interact with each other. The release of inflammatory factors promotes an increase in AGP [46]. AGP has also been found to be selectively induced in adipose tissue of obese mice to suppress excessive inflammation [47]. AGP and CAP were negatively correlated when AGP > 1.2 g/L. We hypothesize that AGP binds to inositol 1, 4, 5-trisphosphate receptor type 2 to activate AMP-activated protein kinase signaling, which inhibits sterol regulatory element binding protein 1c-mediated (SREBP1c) lipogenic gene program [12,48]. AGP can suppress further development of NAFLD by inhibiting the SREBP1 pathway [49]. Therefore, this finding suggests that AGP may serve as a potential therapeutic target for NAFLD and fibrosis treatment.

Subgroup analysis found that the association between AGP and NAFLD was significantly different between diabetic and non-diabetic populations. NAFLD patients usually exhibit abnormal glucose metabolism. Their risk of type 2 diabetes (T2DM) has tripled [50]. The regression of hepatic steatosis can prevent the onset of T2DM [51]. Recent evidence suggests that T2DM is an independent risk factor for NAFLD [52]. IR is one of the key events that co-occur in NAFLD and diabetes [53]. Studies have shown that AGP is associated not only with IR in adipose tissue and adiponectin but also with a family history of T2DM [54]. In addition, SREBP1c can enhance the production of harmful lipid molecules, such as diacylglycerol and ceramide, thereby further enhancing IR [55]. AGP may play a role by affecting SREBP1c and IR.

Future research should focus on several key directions: first, longitudinal studies are needed to establish whether AGP changes precede, coincide with, or follow liver disease progression. Second, mechanistic studies exploring how AGP interacts with lipid metabolism and fibrogenesis pathways, particularly in the context of diabetes, could uncover potential therapeutic targets. Third, intervention studies examining whether lifestyle or pharmacological interventions that affect AGP levels or glycosylation patterns also impact liver disease outcomes would be valuable. Finally, exploring the relationship between AGP gene polymorphisms and NAFLD/LF susceptibility could provide insights into genetic risk factors.

This study has several limitations. First, the cross-sectional design does not allow for a clear causal relationship. Second, we could not fully exclude the interference of additional confounding factors, such as medication use. In our study, we used VCTE to assess NAFLD and LF. Despite its high accuracy, VCTE still could not be fully consistent with biopsy results [56,57]. In addition, while this study examined AGP levels, it did not focus on the altered glycosylation patterns of AGP. Further studies are needed to uncover the connection. Nevertheless, this study has several strengths. As the first cross-sectional study focusing on AGP and chronic liver lesions in adult women, our study included a nationally representative population. Furthermore, our subgroup analysis yielded significant and consistent findings.

## 5. Conclusions

Overall, we demonstrated a significant positive correlation between AGP and both NAFLD and LF in adult females, with odds ratios of 12.00 [95% CI (6.73, 21.39)] and 2.20 [95% CI (1.07, 4.50)], respectively, after adjusting for multiple covariates. Additionally, we identified an inverted U-shaped relationship between AGP and CAP with an inflection point at 1.20 g/L. Subgroup analysis revealed that the association between AGP and NAFLD was significantly different in the diabetic subgroup (*p* < 0.05 for interaction). These findings suggest the potential utility of AGP as a non-invasive diagnostic marker for NAFLD and LF and highlight the complex role of AGP in liver disease pathophysiology.

## Figures and Tables

**Table 1:** Weighted characteristics of the study population based on CAP or LSM.

	Non-NAFLD (CAP < 274, n = 1563)	NAFLD (CAP ≥ 274, n = 707)	*p* Value	Normal Group (LSM < 8.0, n = 2121)	LF (LSM ≥ 8.0, n = 149)	*p* Value
Age (years)	34.43 (33.84, 35.01)	34.28 (33.23, 35.33)	0.807	34.32 (33.78, 34.87)	35.11 (33.17, 37.06)	0.432
Race/Ethnicity (%)			0.988			0.745
Non-Hispanic White	601 (56.17%)	275 (55.17%)		816 (55.64%)	60 (58.72%)	
Non-Hispanic Black	302 (10.73%)	138 (10.95%)		406 (10.73%)	34 (11.65%)	
Mexican American	197 (9.88%)	94 (10.21%)		273 (10.01%)	18 (9.68%)	
Other Race	463 (23.22%)	200 (23.67%)		626 (23.62%)	37 (19.95%)	
Education level (%)			0.064			0.711
<High school	211 (9.60%)	78 (6.17%)		275 (8.66%)	14 (6.71%)	
High school	284 (20.41%)	116 (18.98%)		368 (19.74%)	32 (22.88%)	
College or above	1068 (69.99%)	513 (74.85%)		1478 (71.60%)	103 (70.41%)	
Marital status			0.631			0.533
Married/Living with partner	857 (61.21%)	404 (63.30%)		1189 (61.48%)	90 (66.85%)	
Widowed/Divorced/Separated	200 (9.63%)	88 (10.01%)		273 (9.89%)	15 (7.94%)	
Never married	488 (29.17%)	215 (26.69%)		659 (28.63%)	44 (25.21%)	
PIR	2.89 (2.77, 3.01)	2.91 (2.74, 3.07)	0.828	2.88 (2.76, 2.99)	3.09 (2.83, 3.36)	0.127
BMI, kg/m^2^	29.26 (28.72, 29.80)	30.37 (29.35, 31.40)	0.056	29.42 (28.94, 29.91)	32.03 (29.93, 34.14)	0.017
Smoked at least 100 cigarettes (%)	377 (24.54%)	223 (31.53%)	0.008	551 (26.14%)	49 (34.48%)	0.141
Diabetes (%)	41 (2.46%)	128 (19.55%)	<0.001	126 (5.99%)	43 (31.83%)	<0.001
Hypertension (%)	382 (23.79%)	258 (35.93%)	<0.001	578 (26.61%)	62 (40.57%)	0.007
History of CVD (%)	22 (0.91%)	23 (3.79%)	<0.001	36 (1.39%)	9 (7.37%)	<0.001
Laboratory data						
Total Cholesterol (mmol/L)	4.72 (4.66, 4.79)	4.70 (4.60, 4.79)	0.565	4.70 (4.64, 4.75)	4.95 (4.76, 5.14)	0.010
LSM (kPa)	4.52 (4.44, 4.61)	6.16 (5.73, 6.58)	<0.001	-	-	-
CAP (dB/m)	-	-	-	241.47 (238.13, 244.81)	311.79 (298.61, 324.96)	<0.001
AGP (g/L)			<0.001			0.027
Tertile 1 (<0.673)	650 (43.04%)	107 (15.60%)		728 (35.45%)	29 (21.28%)	
Tertile 2 (0.673–0.879)	524 (31.50%)	231 (34.06%)		715 (32.49%)	40 (29.76%)	
Tertile 3 (>0.879)	389 (25.46%)	369 (50.33%)		678 (32.06%)	80 (48.97%)	

Notes: All estimates accounted for complex survey designs, and all percentages are weighted. NAFLD, nonalcoholic fatty liver disease; CAP, controlled attenuation parameter; LF, liver fibrosis; LSM, liver stiffness measure; PIR, ratio of family income to poverty; BMI, body mass index; CVD, cardiovascular disease; AGP, alpha-1-acid glycoprotein.

**Table 2:** The association between AGP and NAFLD.

	Model 1 OR (95% CI)	*p* Value	Model 2 OR (95% CI)	*p* Value	Model 3 OR (95% CI)	*p* Value
AGP, continuous	14.95 (8.42, 26.53)	<0.001	15.08 (8.46, 26.88)	<0.001	12.00 (6.73, 21.39)	<0.001
AGP, in tertiles						
Tertile 1 (<0.673)	reference		reference		reference	
Tertile 2 (0.673–0.879)	2.98 (2.11, 4.21)	<0.001	2.98 (2.12, 4.20)	<0.001	2.76 (1.98, 3.84)	<0.001
Tertile 3 (>0.879)	5.45 (4.05, 7.33)	<0.001	5.49 (4.07, 7.41)	<0.001	4.87 (3.67, 6.45)	<0.001
*p* for trend		<0.001		<0.001		<0.001

Notes: Model 1: No covariates were adjusted. Model 2: Age and race were adjusted. Model 3: Age, race, educational level, marital status, PIR, BMI, smoking, diabetes, hypertension, history of CVD, and total cholesterol were adjusted.

**Table 3:** The association between AGP and LF.

	Model 1 OR (95% CI)	*p* Value	Model 2 OR (95% CI)	*p* Value	Model 3 OR (95% CI)	*p* Value
AGP, continuous	3.62 (1.67, 7.85)	0.002	3.64 (1.68, 7.89)	0.002	2.20 (1.07, 4.50)	0.042
AGP, in tertiles						
Tertile 1	reference		reference		reference	
Tertile 2	1.53 (0.66, 3.52)	0.329	1.52 (0.66, 3.52)	0.334	1.27 (0.56, 2.89)	0.580
Tertile 3	2.54 (1.25, 5.19)	0.014	2.56 (1.25, 5.28)	0.015	1.90 (0.97, 3.72)	0.075
*p* for trend		<0.001		<0.001		<0.001

Notes: Model 1: No covariates were adjusted. Model 2: Age and race were adjusted. Model 3: Age, race, educational level, marital status, PIR, BMI, smoking, diabetes, hypertension, history of CVD, and total cholesterol were adjusted.

**Table 4:** Threshold effect analysis of AGP levels on CAP using a two-piecewise linear regression model.

AGP	CAP Adjusted β (95% CI)	*p* Value
Fitting by the standard linear model	82.69 (73.10, 92.28)	<0.001
Fitting by the two-piecewise linear model		
Inflection point	1.20	
<K segment effect	102.26 (91.46, 113.06)	<0.001
>K segment effect	−60.07 (−99.09, −21.05)	0.003
Log likelihood ratio		<0.001

**Table 5:** Subgroup analysis of the association between AGP with NAFLD and LF.

Subgroup	NAFLD OR (95% CI)	*p* for Interaction	LF OR (95% CI)	*p* for Interaction
Race		0.535		0.649
Non-Hispanic White	10.06 (3.60, 28.10)		2.92 (1.17, 7.29)	
Non-Hispanic Black	16.56 (4.20, 65.21)		0.75 (0.11, 5.16)	
Mexican American	7.07 (1.62, 30.82)		1.81 (0.54, 6.08)	
Other Race	20.37 (9.24, 44.88)		1.67 (0.22, 12.51)	
Education level (%)		0.331		0.036
<High school	3.66 (0.82, 16.45)		1.35 (0.21, 8.67)	
High school	11.36 (2.84, 45.43)		0.43 (0.08, 2.28)	
College or above	13.60 (6.59, 28.03)		3.43 (1.68, 7.01)	
Marital status		0.942		0.020
Married/Living with partner	11.06 (4.69, 26.09)		1.83 (0.86, 3.92)	
Widowed/Divorced/Separated	14.88 (2.90, 76.39)		18.17 (4.81, 68.61)	
Never married	13.51 (6.13, 29.80)		2.04 (0.35, 11.88)	
PIR		0.546		0.147
PIR ≤ 1.3	21.01 (8.41, 52.45)		10.00 (1.51, 66.19)	
1.3 < PIR ≤ 3.5	10.07 (3.74, 27.14)		2.39 (0.84, 6.83)	
PIR > 3.5	11.35 (3.46, 37.24)		1.21 (0.38, 3.86)	
BMI, kg/m^2^		0.056		0.492
BMI < 25	8.18 (2.67, 25.04)		1.61 (0.52, 4.98)	
25 ≤ BMI < 30	39.39 (13.68, 113.41)		6.83 (0.69, 67.87)	
BMI ≥ 30	8.58 (3.91, 18.81)		1.75 (0.73, 4.20)	
Smoked at least 100 cigarettes (%)		0.188		0.538
Yes	21.24 (8.84, 51.04)		1.53 (0.43, 5.41)	
No	9.79 (4.77, 20.07)		2.49 (1.06, 5.85)	
Diabetes (%)		0.011		0.349
Yes	0.75 (0.10, 5.85)		0.86 (0.09, 8.20)	
No	14.41 (7.50, 27.70)		2.76 (1.27, 5.99)	
Hypertension (%)		0.235		0.547
Yes	6.97 (2.35, 20.73)		1.53 (0.40, 5.90)	
No	15.09 (7.64, 29.79)		2.54 (1.08, 5.94)	
History of CVD (%)		0.736		0.658
Yes	43.20 (0.02, 78,603.52)		3.45 (0.56, 21.37)	
No	11.73 (6.53, 21.09)		2.09 (0.90, 4.85)	
Total Cholesterol (mmol/L)		0.852		0.220
≤6	11.99 (6.56, 21.93)		2.79 (1.35, 5.78)	
>6	10.37 (2.36, 45.57)		0.76 (0.12, 4.88)	

Notes: The results were adjusted for all covariates except for the corresponding stratification variable.

## Data Availability

Publicly available datasets were analyzed in the present study. All detailed data can be found here: www.cdc.gov/nchs/nhanes/ (accessed on 10 October 2024).

## Abbreviations

AFP	Alpha-fetoprotein
AGP	Alpha-1-acid glycoprotein
BMI	Body mass index
CAP	Controlled attenuation parameter
CVD	Cardiovascular disease
HCC	Hepatocellular carcinoma
LF	Liver fibrosis
LSM	Liver stiffness measurement
NAFLD	Nonalcoholic fatty liver disease
NASH	Non-alcoholic steatohepatitis
NHANES	National Health and Nutrition Examination Survey
PIR	Ratio of family income to poverty
SREBP1c	Sterol regulatory element binding protein 1c
VCTE	Vibration-controlled transient elastography
